# A Leakage-Aware Drug Discovery Workflow for PKM2 and MAPK1 Integrating Scaffold Validation, Molecular Docking and Structural Triage

**DOI:** 10.3390/ijms27114751

**Published:** 2026-05-25

**Authors:** Ferhat Ucar, Nida Kati

**Affiliations:** 1 Software Engineering Department, Faculty of Technology, Fırat University, Elazığ 23200, Turkey; fucar@firat.edu.tr; 2 Metallurgical and Materials Engineering Department, Faculty of Technology, Fırat University, Elazığ 23200, Turkey

**Keywords:** computer-aided drug discovery, virtual screening, PKM2, MAPK1, LIT-PCBA, scaffold split, uncertainty calibration, ADMET, molecular docking

## Abstract

Computer-aided drug discovery increasingly depends on virtual-screening workflows that remain reliable under severe class imbalance, chemical redundancy and early-recognition constraints. In this study, we developed a leakage-aware prioritization workflow for two cancer-relevant targets, pyruvate kinase M2 (PKM2) and mitogen-activated protein kinase 1 (MAPK1/ERK2), using the LIT-PCBA benchmark. The workflow combines canonical-SMILES curation, duplicate and label-conflict auditing, scaffold-aware validation, a non-learning nearest-active Tanimoto baseline, imbalance-aware machine-learning models, repeated-seed robustness analysis, isotonic probability calibration, ensemble-disagreement estimation, absorption, distribution, metabolism, excretion and toxicity (ADMET)-aware triage, molecular docking, and residue-level contact analysis. Benchmark enrichment is interpreted alongside calibration, ADMET filtering, docking and residue-contact evidence, rather than as a standalone discovery claim. PKM2 emerged as the clearer machine-learning case, with scaffold-aware tree models improving early recognition beyond the nearest-active similarity baseline and yielding top-ranked candidates supported by calibrated activity scores, ADMET profiles, docking scores, and residue-contact fingerprints. MAPK1 provided a biologically relevant contrast target, where ligand-neighborhood similarity remained competitive and downstream structural triage became more decisive than ligand-based ranking alone. These results support a conservative drug-discovery workflow in which leakage-aware benchmarking, calibration, uncertainty, and molecular-level triage remain visible throughout candidate prioritization.

## 1. Introduction

Computer-aided drug discovery (CADD) has become a practical way to reduce large chemical libraries before experimental testing. In parallel, virtual screening has expanded from straightforward similarity filtering to a broader landscape that includes machine learning, deep learning and hybrid workflows combining ligand-based and structure-based evidence. These developments create new opportunities for candidate prioritization, but they also make careful evaluation more important [1,2,3]. Recent large-scale analyses have further questioned how well widely used molecular fingerprints can recover chemically diverse actives on their own in very large search spaces, so simple similarity performance is better treated as a necessary benchmark than as a direct proxy for discovery value [4]. This issue is particularly relevant in cancer-focused screening, where useful targets often sit inside dense metabolic and signalling networks and candidate ranking must balance biological relevance, chemical novelty, pharmacokinetic risk and structural plausibility. Two targets that reflect this challenge are pyruvate kinase M2 (PKM2), a regulator of cancer metabolism, and mitogen-activated protein kinase 1 (MAPK1/ERK2), a central kinase in oncogenic signalling.

Despite this progress, virtual-screening benchmarks are still easy to overread. A model can achieve a strong global discrimination score while still failing where screening matters most, namely at the very top of the ranked list. Random train–test splits can also be overly generous when close analogues appear in both partitions. In highly imbalanced screening problems, the key question is therefore not whether a classifier separates all actives from all inactives in aggregate, but whether it can place a small number of true actives early enough to support follow-up. For that reason, enrichment factor, precision–recall behaviour, BEDROC-like early-recognition scores, scaffold-aware validation and calibration quality are more informative here than accuracy alone [5].

The LIT-PCBA benchmark was created to provide more realistic ligand-based virtual-screening tasks than artificial decoy collections [6]. Even so, practical guidance and broader review work both show that benchmark discipline, data quality and score interpretation matter at least as much as algorithm choice when screening results are expected to inform medicinal chemistry [7,8]. Exact duplicates, label conflicts and chemical-neighborhood leakage can still distort the picture. A model that looks highly predictive under a random split may be doing little more than recovering near-neighbor similarity, and recent work suggests that even scaffold splits can remain optimistic when benchmark design is not examined carefully enough [9,10]. For that reason, a fair study should compare machine-learning outputs not only against one another, but also against a deliberately simple maximum-similarity-to-training-actives baseline.

Here, we use PKM2 and MAPK1 as complementary case studies to build a more conservative CADD workflow. PKM2 is a strong cancer-metabolism target for this purpose because its role extends beyond glycolytic flux toward broader proliferative rewiring, transcriptional regulation and tumor-supporting metabolic plasticity [11,12]. It also has a recognizable CADD literature that includes structure-based screening, docking-led inhibitor selection, natural-product-focused inhibitor discovery and more recent repurposing-oriented multi-step screening [13,14,15]. A recent medicinal chemistry review covering small-molecule PKM2 inhibitor development from 2020 to 2025 further supports the view that PKM2 remains an active target space in which computational triage needs to be connected to chemically interpretable candidate selection [16]. MAPK1/ERK2 provides a useful signalling-pathway contrast: ERK-centred MAPK signalling remains deeply involved in malignant proliferation, survival and progression across tumour types, yet it also represents the pathway-level complexity that often makes clean screening generalization difficult [17,18]. Recent pathway-level reviews of BRAF/MAPK targeting across cancers likewise emphasize that therapeutic relevance in this signalling axis does not remove the need for careful molecular and context-aware interpretation [19]. Recent ERK2-focused studies likewise rely on multi-stage computational filtering, including integrated in silico and in vitro screening for ERK2 dimerization inhibitors [20,21]. Against that background, our study is not framed as a simple classifier contest. Instead, it asks whether calibrated, uncertainty-aware ensemble learning adds useful ranking value beyond nearest-active similarity under scaffold-aware validation, and whether the resulting candidates remain plausible after absorption, distribution, metabolism, excretion and toxicity (ADMET) filtering, molecular docking and molecular-level structural analysis. The broader aim is to connect benchmark discipline with practical hit triage while treating uncertainty as an interpretable screening signal rather than a decorative model output and by keeping molecular evidence visible at the final prioritization stage [22,23].

The main contributions of this work are as follows:

- A target-level curation and leakage audit of PKM2 and MAPK1 LIT-PCBA subsets;
- A direct comparison between nearest-active similarity and imbalance-aware machine-learning baselines under random and scaffold splits;
- Repeated-seed robustness analysis using early-recognition metrics rather than accuracy;
- Isotonic calibration and ensemble-disagreement estimates for uncertainty-aware ranking;
- ADMET-aware and molecular-docking-aware prioritization of scaffold-diverse candidates; and
- Residue-level contact fingerprints as a molecular-level check that top docking poses produce coherent target-specific interaction patterns.

**Figure 1:**
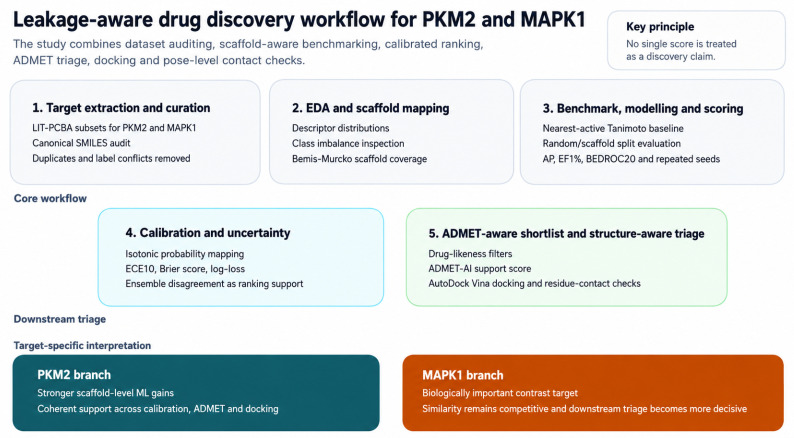
Overview of the leakage-aware drug discovery workflow used for PKM2 and MAPK1. The figure summarizes the staged design of the study, from target extraction and curation to scaffold-aware benchmarking, score calibration, ADMET filtering and structure-aware triage. PKM2 and MAPK1 are retained as complementary target branches so that target-specific behavior can be interpreted directly rather than averaged away in a pooled analysis.

These elements place the study in a specific part of the current CADD literature. We do not argue that a stronger benchmark score, by itself, is enough to support a discovery claim. Instead, we show how leakage-aware benchmarking, explicit similarity baselines, calibration, uncertainty and downstream triage can be combined into a more disciplined ranking workflow for realistic ligand-based screening tasks. The analytical sequence is summarized in Figure 1.

As shown in Figure 1, the study is organized as a staged prioritization pipeline rather than a single-model exercise. The workflow begins with target extraction, curation and exploratory scaffold mapping, then moves to benchmark comparison and score calibration, and only then proceeds to ADMET filtering, docking and target-specific interpretation. This order is intentional: each downstream step is used to refine a chemically plausible shortlist, not to rescue weak ligand-based ranking signals.

The methodological positioning of the study is therefore deliberately conservative. The novelty is not that scaffold splitting, calibration, ADMET prediction, docking or contact analysis are individually new. Recent leakage-aware studies, including the work of Guo and co-workers, already make clear that split design and chemical-neighborhood structure can strongly affect virtual-screening conclusions [9,10]. Our contribution is to carry that warning into an auditable candidate-prioritization workflow: every shortlisted molecule is interpreted through an explicit similarity baseline, scaffold-aware model behaviour, calibrated confidence, ensemble disagreement, predicted ADMET risk, docking support and residue-level pocket contacts. This makes the output a traceable decision chain rather than a single benchmark number or a docking-only hit list. In that sense, the manuscript is both an applied PKM2/MAPK1 study and a methodological statement about how prospective-looking virtual-screening claims should be supported.

The remainder of the manuscript is organized as follows. Section 2 presents the dataset audit, benchmark comparisons, robustness analysis, calibration outcomes and multi-stage candidate prioritization results. Section 3 interprets the target-specific differences and study limitations. Section 4 then describes dataset curation, exploratory analysis, model development, calibration, ADMET scoring, docking and pose-contact extraction, and Section 5 summarizes the main conclusions.

## 2. Results

The results are presented in the same order as the analytical workflow in Figure 1. We first show how target-level curation and scaffold structure define the difficulty of the screening problem, then compare similarity and machine-learning performance under leakage-aware validation, and then move to calibration, ADMET-aware triage, docking and pose-level contact analysis. This ordering matters because the downstream shortlist is only meaningful when the earlier benchmarking steps are interpreted conservatively.

### 2.1. Dataset Curation Reveals Severe Class Imbalance

**Figure 2:**
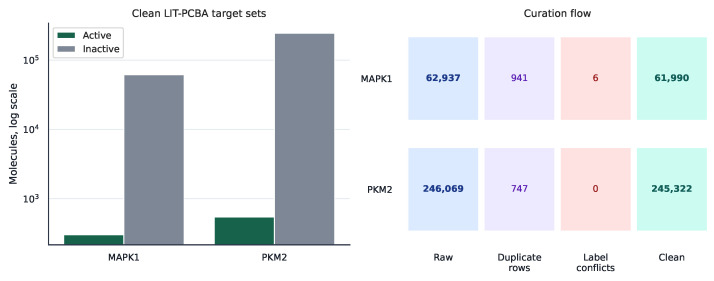
Dataset landscape and curation audit for the PKM2 and MAPK1 LIT-PCBA target sets. The final clean sets retain severe active/inactive imbalance while excluding duplicate and label-conflicting entries. The molecule-count axis is shown on a logarithmic scale to make the active and inactive classes visible in the same panel. This visual summary complements Table 1 by showing the imbalance and curation flow, whereas the table reports the exact numerical counts.

Both LIT-PCBA target sets represent realistic, highly imbalanced virtual-screening problems. PKM2 retained 546 actives among 245,322 cleaned molecules, and MAPK1 retained 302 actives among 61,990 cleaned molecules. Figure 2 summarizes the clean-set imbalance and the curation audit, while Table 1 makes the data reduction explicit at the target level. The table and figure are intended to serve different purposes: Table 1 provides the exact target-level counts needed for reproducibility, whereas Figure 2 gives a visual audit of imbalance and row removal before modelling. The curation effect was modest for PKM2 but non-negligible for MAPK1, where conflicting labels would otherwise have introduced avoidable ambiguity into model evaluation. This first step is not only technical housekeeping; it defines the credibility of everything that follows. The benchmark and calibration settings were kept fixed across targets and seeds as described in the Section 4, so the target-level differences reported below are not artifacts of target-specific retuning.

**Figure 3:**
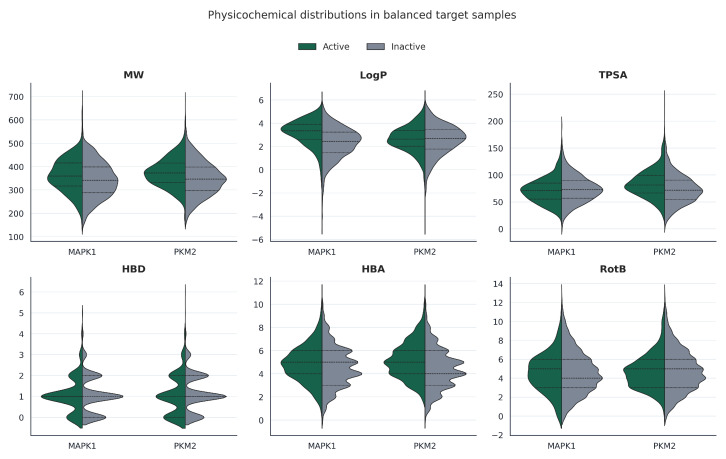
Physicochemical descriptor distributions for active and inactive molecules in balanced target samples.

Descriptor distributions showed that active compounds were not simply arbitrary samples from the inactive pool. MAPK1 actives were somewhat heavier and more lipophilic, whereas PKM2 actives showed higher molecular weight, TPSA and hydrogen-bond acceptor counts. These trends are visible in Figure 3, which helps frame later model behaviour: the targets are separable in descriptor space to some degree, but not in a way that would justify trusting a random split without a chemical-leakage check.

### 2.2. Active Scaffolds Are Diverse Rather than Dominated by a Single Chemotype

**Figure 4:**
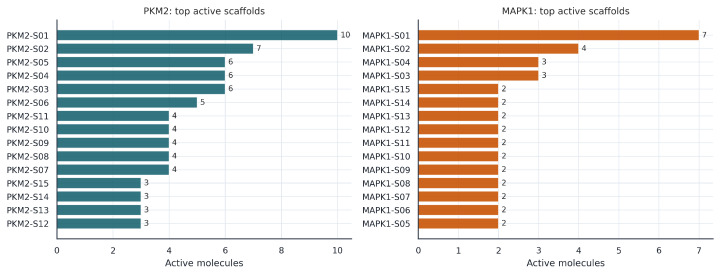
Most frequent active Murcko scaffolds in the cleaned PKM2 and MAPK1 target sets. Scaffold identifiers indicate rank within each target; bar labels report the active-molecule count for each scaffold.

**Figure 5:**
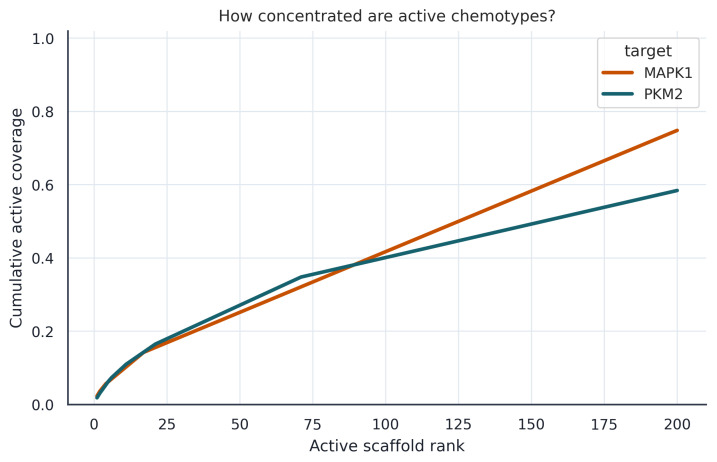
Cumulative active coverage by scaffold rank. The gradual curves indicate that active molecules are distributed over many scaffold families.

Murcko scaffold analysis showed that neither target was dominated by a single active scaffold. The top active scaffold covered only 1.8% of PKM2 actives and 2.3% of MAPK1 actives, whereas the top 100 active scaffolds covered approximately 40.1% and 41.7%, respectively. Figure 4 and Figure 5 therefore support an important interpretive point: these datasets are not driven by one oversized chemotype, so a scaffold-aware benchmark is testing transfer across many smaller families rather than merely punishing one dominant scaffold series.

### 2.3. Machine Learning Must Be Interpreted Against a Similarity Baseline

The maximum-similarity-to-training-actives baseline produced non-trivial early recognition, confirming that chemical-neighborhood recovery can explain part of the screening signal. In PKM2, the similarity baseline dropped sharply from random to scaffold split, with AP decreasing from 0.1020 to 0.0063 and EF1% from 33.92 to 10.89. MAPK1 remained more recoverable under scaffold split, with a scaffold EF1% of 21.15. This already indicates that the two targets should not be narrated in the same way: PKM2 becomes substantially harder once close analogues are separated, whereas MAPK1 retains more neighborhood-driven recoverability.

**Figure 6:**
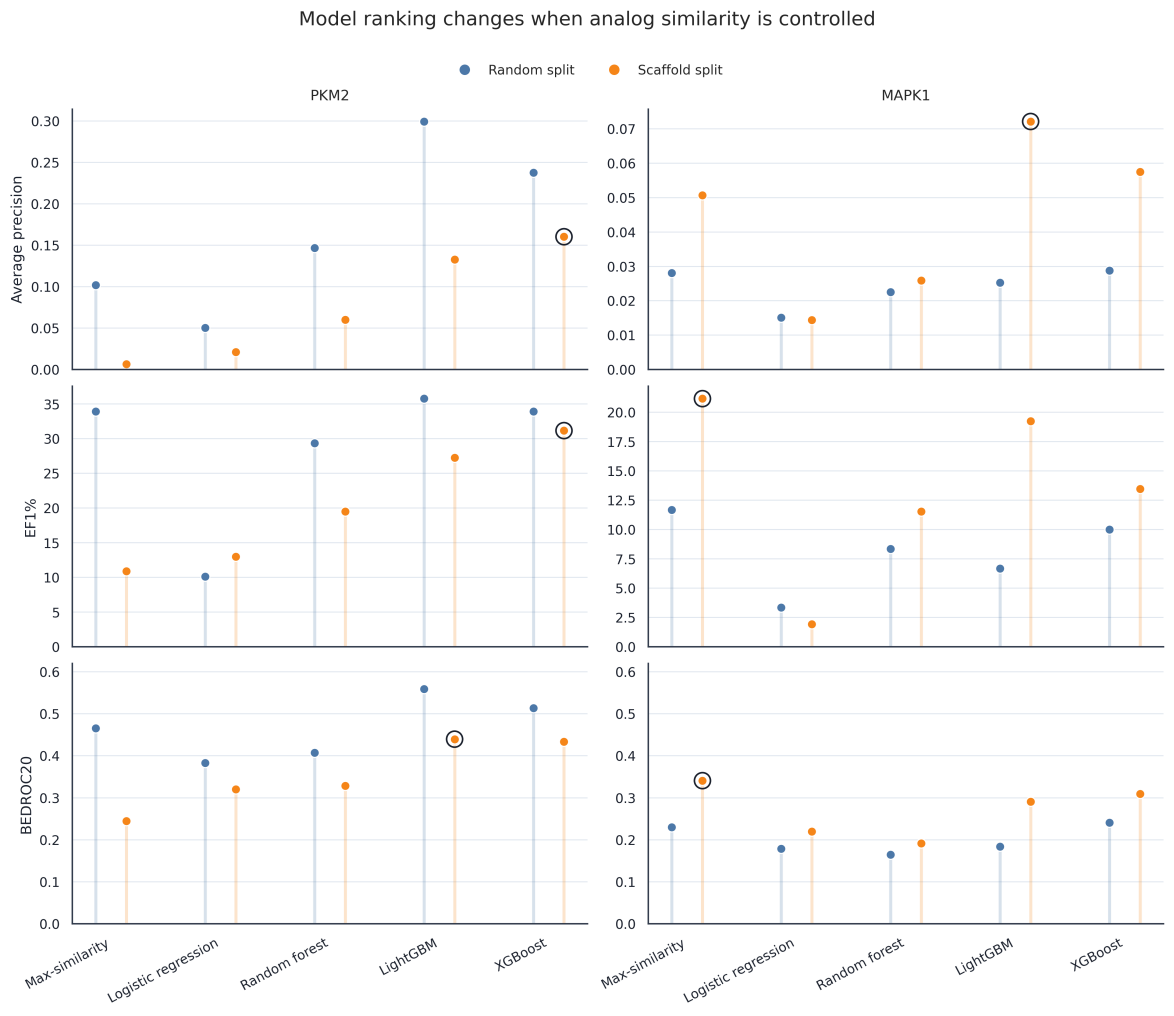
Model evaluation across AP, EF1% and BEDROC20. Similarity and machine-learning models are compared under random and scaffold splits. Blue markers denote random-split results and orange markers denote scaffold-split results. The open circle highlights the best scaffold-split model within each target–metric panel, emphasizing the model that remains strongest after analogue-neighborhood leakage is controlled.

Weighted tree models improved PKM2 early recognition under scaffold-aware evaluation. In the first-pass benchmark, scaffold-split XGBoost achieved AP 0.1603 and EF1% 31.17 for PKM2, substantially above the similarity baseline. MAPK1 was more nuanced: scaffold-split LightGBM achieved AP 0.0722 and EF1% 19.23, close to the similarity baseline. Figure 6 summarizes these random/scaffold and similarity/ML contrasts, and Table 2 gathers the main benchmark numbers in one place. What matters here is not only which model appears strongest, but whether it adds ranking value beyond what can already be recovered from nearest-neighbor chemistry. On that question, PKM2 gives a clear yes, whereas MAPK1 remains a harder case in which ML gains are smaller and should be interpreted more cautiously. For this reason, MAPK1 is interpreted primarily as a biologically relevant contrast target rather than as a second strong ML-success case. Its value in the study is that it shows where ligand-neighborhood similarity remains competitive and where downstream ADMET and structural triage become more important for cautious prioritization. The ECFP6 sensitivity check supported the same interpretation. Under scaffold split, PKM2 retained model-added value with ECFP6, with LightGBM reaching AP = 0.154 and EF1% = 31.17 and XGBoost reaching EF1% = 32.47. MAPK1 did not become a stronger ML case under ECFP6; the best ECFP6 AP was 0.064 and the best EF1% was 15.38. Thus, the PKM2/MAPK1 contrast was not an artefact of using ECFP4 alone, although broader representation studies with descriptor ensembles, MACCS keys or graph neural networks remain outside the scope of the present workflow-focused study. Because ECFP4 and ECFP6 are closely related circular fingerprints, this sensitivity check should be interpreted as a local representation check; a non-circular representation would provide a stronger test of representation dependence. The full-data PKM2 scaffold-split check gave a more conservative absolute scale, as expected when the inactive pool is restored and test-set prevalence decreases. The nearest-active similarity baseline reached AP = 0.0063, EF1% = 10.89 and BEDROC20 = 0.245. On the same full-data split, LightGBM reached AP = 0.0069, EF1% = 16.33 and BEDROC20 = 0.257, while XGBoost reached AP = 0.0061, EF1% = 14.52 and BEDROC20 = 0.263. Thus, the capped benchmark should be read as a tractable repeated-seed comparison, whereas the full-data check supports the same qualitative PKM2 interpretation for early enrichment and BEDROC20 without overstating AP stability (Supplementary Table S4).

### 2.4. Repeated-Seed Analysis Supports PKM2 as the Stronger ML Target

**Figure 7:**
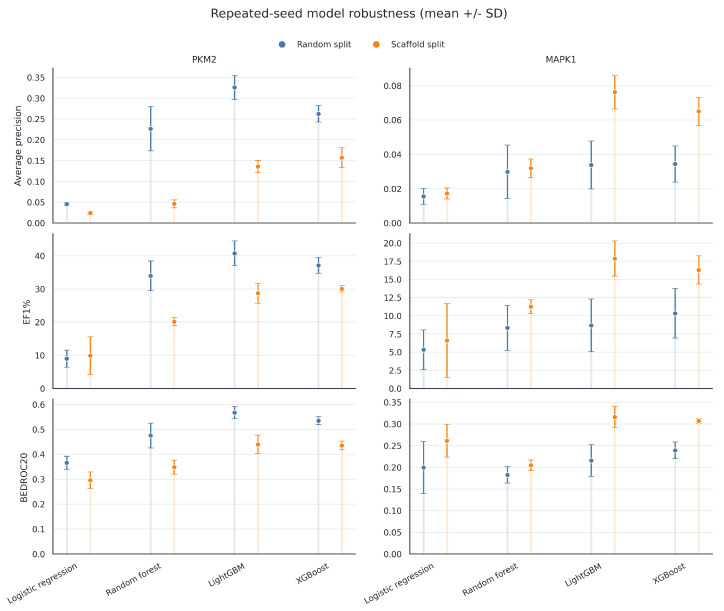
Repeated-seed robustness of ligand-based ML models. PKM2 shows more stable model-added value under scaffold-aware evaluation, whereas MAPK1 remains more tightly coupled to ligand-neighborhood similarity.

Across five seeds, PKM2 remained the more robust machine-learning target. Under random split, LightGBM achieved AP 0.326 ± 0.029, EF1% 40.73 ± 3.70 and BEDROC20 0.568 ± 0.024. Under scaffold split, XGBoost achieved AP 0.157 ± 0.024 and EF1% 30.01 ± 0.97. MAPK1 retained a useful but more modest signal, with scaffold-split LightGBM AP 0.076 ± 0.010 and EF1% 17.86 ± 2.41. Figure 7 shows that this is not a one-seed artefact, and Table 3 clarifies which model remained most reliable for each target and split. In other words, the repeated-seed analysis narrows the manuscript claim: the workflow is strongest for PKM2, whereas MAPK1 behaves more like a contrast case that tests the limits of ligand-based generalization.

### 2.5. Calibration Converts Ranking Scores into More Reliable Prioritization Signals

**Figure 8:**
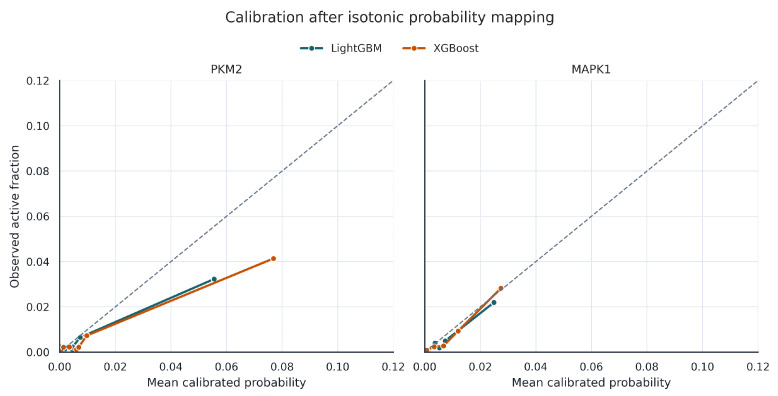
Observed-versus-predicted calibration curves for scaffold-split LightGBM and XGBoost models before and after isotonic calibration. The dotted diagonal line indicates perfect calibration.

**Figure 9:**
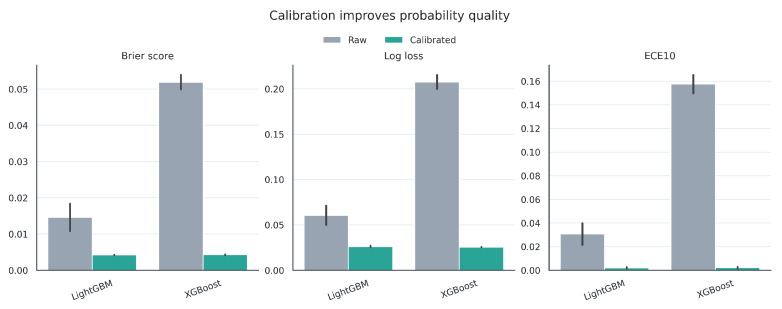
Calibration-related metric changes after isotonic calibration. Lower Brier score, log-loss and ECE10 indicate improved probability quality.

Isotonic calibration substantially improved probability quality. For PKM2, LightGBM ECE10 decreased from 0.0399 to 0.0028 and XGBoost ECE10 decreased from 0.1497 to 0.0028. For MAPK1, LightGBM ECE10 decreased from 0.0214 to 0.0014 and XGBoost ECE10 decreased from 0.1653 to 0.0015. Figure 8 and Figure 9 show that the improvement was not cosmetic: the calibrated scores align much more closely with observed frequencies and reduce probability error across all monitored metrics. This step is central to the manuscript logic because the downstream shortlist is built from interpretable prioritization signals rather than raw classifier margins.

### 2.6. ADMET and Molecular Docking Support a Multi-Objective Shortlist

**Figure 10:**
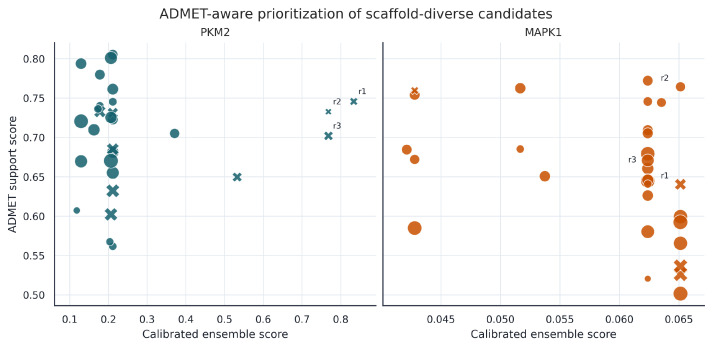
ADMET-aware prioritization of scaffold-diverse candidates. Candidate positions combine calibrated ensemble activity scores with ADMET support scores. Teal and orange markers denote PKM2 and MAPK1 candidates, respectively; marker shape indicates the retrospective LIT-PCBA label, marker size reflects toxicity burden, and r1–r3 mark the top three ADMET-priority ranks within each target. Target-specific x-axis limits are used to preserve within-target readability. The narrow MAPK1 x-axis is not a plotting artefact: the MAPK1 calibrated ensemble scores in this scaffold-diverse ADMET subset span only 0.042–0.065, whereas PKM2 spans 0.118–0.834.

ADMET-aware prioritization retained PKM2 as the cleaner computational branch. In the scaffold-diverse top-30 shortlist, PKM2 included nine known actives, median QED 0.809, median ADMET support 0.722 and median toxicity burden 0.408. MAPK1 included four known actives, median QED 0.708, median ADMET support 0.656 and median toxicity burden 0.479. Figure 10 shows the activity–ADMET trade-off for both targets, and it makes the asymmetry visually clear: PKM2 offers several candidates that stay in a favorable part of both axes, whereas MAPK1 candidates occupy a much narrower activity range and are differentiated more by ADMET support than by ligand-based score alone. These ADMET-derived values are interpreted only as prioritization filters. They help identify candidates that may deserve lower or higher follow-up priority, but they do not establish real pharmacokinetic behaviour, organ-level toxicity, cellular safety or in vivo tolerability. In practice, this is where the two target stories begin to separate most visibly.

To show this contrast on an absolute scale, a shared-x-axis version of the same ADMET prioritization plot is provided as Supplementary Figure S1.

**Figure 11:**
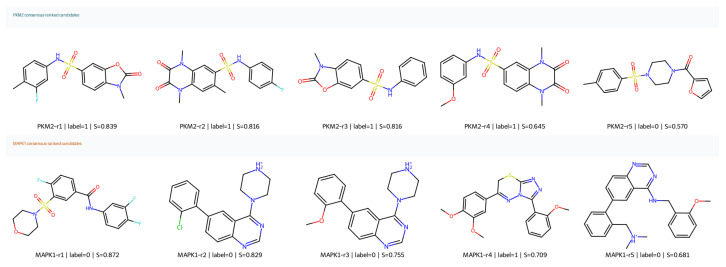
Two-dimensional structures of the top five consensus-ranked candidates for PKM2 and MAPK1. Candidate labels report the retrospective LIT-PCBA label and final consensus score. Atom colors follow standard molecular drawing conventions. The structures make the chemical identity of the prioritized molecules explicit and help distinguish the stronger PKM2 candidate branch from the more cautious MAPK1 branch.

**Figure 12:**
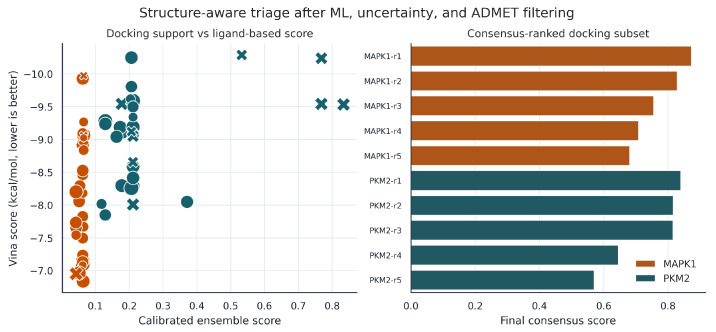
Structure-aware triage after ML, uncertainty and ADMET filtering. The panel summarizes the expanded docked top-30 candidate set per target; the final consensus score combines calibrated activity, ADMET support, toxicity burden and docking support. In the left panel, teal and orange markers denote PKM2 and MAPK1 candidates, marker shape indicates the retrospective LIT-PCBA label, and marker size reflects ADMET support.

Molecular docking further supported the PKM2 branch. To reduce final-ranking ambiguity, the full scaffold-diverse top-30 ADMET-prioritized candidates for each target were docked, and the consensus ranking was recomputed from calibrated activity, ADMET support, toxicity burden and Vina docking support. The top PKM2 consensus candidates combined high calibrated activity scores, acceptable ADMET support and favourable Vina scores. For example, PKM2-r1 had a calibrated activity score of 0.834, ADMET support of 0.746 and Vina score of −9.534 kcal/mol, whereas PKM2-r2 had Vina score of −10.240 kcal/mol. The first four PKM2 consensus-ranked candidates were known actives in the retrospective LIT-PCBA labels. MAPK1 behaved differently: its top-ranked docked candidates remained in a very narrow calibrated-score range of 0.062–0.065, and the first known active appeared at consensus rank 4. This is why MAPK1 is interpreted as a contrast target rather than as an equally strong candidate-discovery branch. Figure 11 shows the 2D structures of the top five consensus-ranked molecules for each target, and Figure 12 shows the shift from ligand-level confidence to molecular-level structural triage. Table 4 summarizes the final consensus-ranked docking subset that carries the main narrative of the paper. The corresponding canonical SMILES and consensus-ranking quantities for the top five candidates per target are provided in Supplementary Table S2, and the complete docked top-30 lists are provided in Supplementary Table S5. As a qualitative weight check, exchanging the activity and ADMET weights preserved the same top-three candidate set for PKM2 and kept the MAPK1 top-three order unchanged; increasing the docking weight mainly changed the internal order within the same PKM2 top-five set. Thus, the main PKM2/MAPK1 interpretation was not driven by a single fragile choice of consensus weights, although the consensus score should still be read as a prioritization heuristic rather than an optimized objective function. Two points matter here. First, the structural layer did not overturn the ligand-based ranking logic; it refined it by separating candidates that were otherwise close in calibrated score. Second, the contrast between PKM2 and MAPK1 remained visible after docking, which argues against a purely cosmetic role for the molecular analysis. PKM2 therefore yields a shortlist supported by multiple independent computational signals, including molecular docking support, while MAPK1 remains more dependent on downstream filtering than on strong ligand-based separation.

The PKM2 shortlist is dominated by compact arylsulfonamide and heteroaryl-sulfonamide chemotypes, with benzoxazinone/imide-like heterocycles appearing among the highest-ranked known actives. This is consistent with the broader structure-based PKM2 screening literature, where drug-like heteroaromatic and sulfonamide-containing small molecules have repeatedly appeared in computational hit-selection contexts [13,14,15]. The MAPK1 shortlist is less decisive: several top-ranked molecules contain kinase-like fused aromatic or quinazoline/heteroaromatic features, but their calibrated activity scores remain narrowly compressed and several carry retrospective inactive labels. We therefore avoid presenting them as novel ERK2 inhibitor scaffolds; instead, they serve as chemically explicit examples of why MAPK1 required downstream structural triage and conservative interpretation [20,21].

### 2.7. Docking Poses Provide Target-Specific Molecular Contact Fingerprints

**Figure 13:**
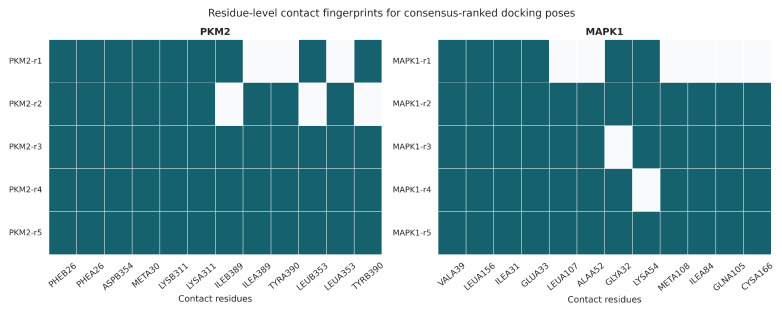
Residue-level contact fingerprints for consensus-ranked docking poses. Contacts were extracted from the first Vina pose using a 4.5 Å residue-contact threshold.

Residue-level contact fingerprints indicated that top-ranked docking poses occupied the intended pockets and produced recurrent target-specific contact patterns. MAPK1 consensus candidates repeatedly contacted GLUA33, VALA39, ILEA31, LEUA156 and polar residues around LYSA54, META108 and ASPA167. PKM2 consensus candidates showed recurrent contacts involving LYSB311/LYSA311 and ASPB354, together with aromatic or hydrophobic contacts around PHEA26/PHEB26 and TYRA390/TYRB390. Figure 13 makes the molecular-evidence layer of the workflow concrete: the top poses are not just numerically well ranked, but also converge onto chemically sensible residue neighborhoods. To make this pattern more explicit across the top consensus ligands, the most frequent pocket contacts for each target were additionally summarized in Supplementary Table S1. These contact patterns also map onto recognizable pocket features. In PKM2, the recurrent contacts with LYS311, ASP354, TYR390, PHE26, MET30, LEU353 and ILE389 place the prioritized poses in the ligand-defined allosteric activator pocket used by the LIT-PCBA 4G1N setup, with a polar rim involving LYS311/ASP354 and an aromatic or hydrophobic wall involving PHE26, TYR390, LEU353 and ILE389. In MAPK1/ERK2, the repeated contacts with LYS54, GLN105, ASP106, MET108 and ASP167 are consistent with occupation of the kinase pocket region sampled by the 4QTE setup: LYS54 is the conserved catalytic lysine, MET108 lies in the hinge region, ASP106/GLN105 are adjacent to the hinge/gatekeeper neighborhood, and ASP167 belongs to the DFG-region side of the ATP-site architecture. These MAPK1 assignments were rechecked against the deposited 4QTE chain-A residue numbering used in the LIT-PCBA receptor file. This residue mapping supports the use of contact fingerprints as a structural sanity check, while still falling short of experimental binding-mode validation. Even so, these fingerprints are used here as a pose-sanity layer, not as experimentally validated binding-mode evidence.

## 3. Discussion

The results show that PKM2 and MAPK1 do not behave in the same way under a shared leakage-aware virtual-screening workflow, and that contrast is one of the main strengths of the manuscript. PKM2 provides the clearest case for model-added value: under scaffold-aware evaluation, tree-based models consistently improved early recognition beyond a nearest-active similarity baseline, calibrated probabilities remained stable enough for downstream ranking and the final candidates retained coherent support from ADMET profiling and docking. This behavior is also biologically plausible. PKM2 has repeatedly been described as a multifunctional regulator that links glycolytic control, non-metabolic signalling and tumor-promoting adaptation, which makes it a reasonable setting for a prioritization workflow that combines ligand-based and structure-based evidence rather than relying on a single score family [11,12]. Earlier PKM2 discovery studies were often organized around docking-led or structure-based screening campaigns aimed at identifying inhibitors or natural-product-like modulators for follow-up [13,14]. More recent work has also moved toward integrated screening and repurposing logic in glioblastoma, which is close in spirit to our effort to make downstream triage explicit rather than treating one docking or ML score as self-sufficient [15]. This positioning is also consistent with recent PKM2 inhibitor-focused review literature, which describes a rapidly developing small-molecule landscape and underlines the need to connect computational prioritization with chemically meaningful inhibitor-selection criteria [16]. Our results therefore sit beside that literature rather than repeating it: the contribution here is not another isolated docking-first screen, but a stricter ranking workflow in which similarity recovery, scaffold transfer, calibration quality, uncertainty and downstream triage are all exposed before any candidate is presented as noteworthy.

MAPK1 remains biologically important and structurally tractable, but its ligand-based signal appears more tightly coupled to chemical-neighborhood recovery. That more cautious outcome is not hard to reconcile with the biology. Reviews of ERK/MAPK signalling emphasize broad context dependence, feedback regulation and tumour-specific wiring, all of which can make a kinase target highly relevant while still limiting straightforward transfer from local ligand similarity to robust scaffold-level ranking [17,18]. A recent review of BRAF/MAPK pathway targeting across cancers reinforces this point at the pathway level: molecular targeting in this axis is clinically and biologically important, but interpretation remains strongly shaped by pathway context, resistance mechanisms and target-state dependence [19]. That reading is also consistent with recent ERK2-focused virtual-screening studies that rely on layered computational filtering, including integrated in silico selection with in vitro follow-up for ERK2 dimerization inhibitors [20,21]. This contrast strengthens the manuscript because it prevents us from making a one-size-fits-all machine-learning claim. We therefore avoid presenting MAPK1 as an equivalent success branch. Instead, it is retained as a useful stress test for the workflow: when scaffold-level ML gains are modest, the analysis must rely more heavily on similarity context, calibrated uncertainty, predicted ADMET liabilities and molecular-level pose checks before any candidate is prioritized.

The PKM2/MAPK1 contrast also suggests a broader screening lesson. PKM2, as a non-kinase metabolic enzyme with an allosteric ligand-defined pocket in the present setup, may offer a setting where scaffold-level ligand-based learning and pocket-contact triage can reinforce one another more clearly. MAPK1/ERK2, by contrast, is a kinase target sampled through a pocket related to a highly conserved ATP-site architecture, where many ligands share recognizable kinase-like features and local chemical-neighbourhood similarity can remain highly competitive. This does not mean that kinase targets are unsuitable for leakage-aware ML screening, or that allosteric targets are automatically easier. It means that target class, binding-site conservation and ligand-series structure should shape how screening evidence is interpreted. For allosteric or non-kinase pockets, model-added value may be more informative when it survives scaffold separation and agrees with residue-level pocket contacts. For kinase-like targets, the same workflow may be most useful as a guardrail: it helps distinguish genuine model-added ranking value from analogue recovery, and it prevents biologically attractive targets from being overclaimed when the ligand-based signal remains narrow.

A central point of the workflow is that no single metric is allowed to dominate the interpretation. AUROC remains secondary, accuracy is deliberately avoided, and raw model outputs are not treated as probabilities without calibration. Instead, the workflow moves from cleaned data to similarity baselines, scaffold-aware ML, repeated-seed robustness, calibration, uncertainty, ADMET filtering, molecular docking and contact-fingerprint checks. In that sense, the workflow includes both ligand-level ranking and molecular-level structural triage. This layered structure is more conservative than a conventional “train several classifiers and report the best score” study, and it is consistent with both longstanding cautions in virtual screening and more recent warnings that even apparently rigorous split strategies can overstate practical screening performance when chemical redundancy and ranking realism are not examined directly [8,9,10]. It also aligns with a broader shift in chemistry-oriented machine learning, where uncertainty is increasingly treated as part of model trustworthiness and decision-making rather than as an afterthought [22,23]. Our decision to keep a nearest-active similarity baseline visible also follows recent evidence that common fingerprint representations may fail to recover chemically diverse actives at large screening scale, even when they remain attractive as fast first-pass filters [4].

From a workflow-design perspective, this matters because candidate prioritization in realistic discovery settings rarely fails for only one reason. A shortlist can look promising because of analogue leakage, because calibration is poor, because ADMET filtering is ignored, or because a numerically attractive docking score is accepted without any structural sanity check. The present workflow was built to make those failure modes easier to see. That design choice does not make the study experimentally complete, but it does make the computational evidence easier to interpret. In molecular informatics and computer-aided drug discovery, this transparency is part of the contribution rather than just a reporting preference.

The study also has limitations. We have therefore made the boundary of the computational claim explicit. The activity labels are binary outputs inherited from LIT-PCBA, so they do not model potency gradients, assay uncertainty or target-specific mechanistic subclasses. The two datasets are also highly imbalanced, which is why accuracy was avoided as an interpretive metric and the evaluation focused instead on PR-AUC, EF1%, BEDROC20, calibration and scaffold-aware behavior. The ligand-based models were built on ECFP4 fingerprints as the primary representation; this choice keeps the benchmark transparent and directly comparable with the nearest-active similarity baseline, but it also means that representation dependence remains a limitation. For this reason, the final shortlist was interpreted through a multi-criteria lens rather than through the performance of any single model: candidate priority required agreement across leakage-aware ML behavior, similarity context, calibrated confidence, uncertainty, ADMET risk, docking support and residue-contact plausibility. Repeated-seed experiments used an 80,000-row cap even though all actives were retained. Docking was used as a triage layer, not as evidence of binding affinity. This caution is particularly important for MAPK1/4QTE, where the reference-ligand redocking generated a near-native pose among the sampled modes but the top-scoring pose was not near-native, making the MAPK1 docking interpretation conditional on the sampled receptor geometry and pose ranking. That distinction matters, because both methodological reviews and practical screening workflows make clear that docking scores can support prioritization while still requiring cautious interpretation and orthogonal follow-up [7,24]. The ADMET layer should be interpreted with similar care: the toxicity-related outputs were used to deprioritize riskier candidates at the ranking stage, but they do not establish compound safety and cannot substitute for experimental toxicology. The contact fingerprints are likewise heuristic and are intended for pose sanity checking rather than definitive mechanistic assignment. Future work should extend the workflow to external prospective libraries, include manual inspection of the highest-priority poses, test molecular-dynamics stability for the final PKM2 shortlist and, where possible, move toward experimental validation.

## 4. Materials and Methods

### 4.1. Dataset Selection and Curation

The PKM2 and MAPK1 subsets were extracted from the local LIT-PCBA distribution [6]. The raw PKM2 set contained 546 actives and 245,523 inactives, whereas the raw MAPK1 set contained 308 actives and 62,629 inactives. All molecules were parsed and canonicalized with RDKit. Invalid structures, exact duplicate rows and canonical-SMILES-level label conflicts were audited before modelling.

The final conservative clean tables contained 245,322 PKM2 molecules, including 546 actives and 244,776 inactives, and 61,990 MAPK1 molecules, including 302 actives and 61,688 inactives. Relative to the raw extraction, PKM2 lost 747 exact duplicate rows, while MAPK1 lost 941 duplicate rows and six canonical-SMILES-level label conflicts. The conflicting MAPK1 entries were not reassigned to either the active or inactive class. Instead, every row belonging to a canonical SMILES that appeared with both labels was removed before duplicate collapse, so the final MAPK1 table contains only unambiguous canonical-SMILES–label pairs. This preprocessing step was treated as part of the method rather than as a hidden data-cleaning operation.

### 4.2. Exploratory Chemical-Space Analysis

RDKit descriptors were computed for molecular weight, logP, topological polar surface area (TPSA), hydrogen-bond donors and acceptors, rotatable bonds, ring counts and heavy atoms. Bemis–Murcko scaffolds were used to summarize active-scaffold concentration and to support scaffold-aware split construction. The exploratory analysis was used to establish the scale, imbalance and chemical diversity of the two targets before model training.

### 4.3. Similarity Baseline

For each target and split, a non-learning similarity baseline was calculated using Morgan circular fingerprints with radius 2, corresponding to diameter 4 in the original ECFP terminology and therefore denoted here as ECFP4; the fingerprint length was 2048 bits. Each test molecule was scored by its maximum Tanimoto similarity to the active molecules in the corresponding training set. This baseline asks a deliberately simple question: how much early recognition can be explained by proximity to known training actives? Machine-learning models were interpreted only in relation to this baseline.

### 4.4. Machine-Learning Models and Validation

ECFP4 fingerprints were used as ligand representations for initial model development. The benchmark included class-weighted logistic regression, class-balanced random forests, weighted LightGBM [25] and weighted XGBoost [26] models. These models were selected as strong tabular baselines for ligand-based screening, while keeping the benchmark focused on evaluation realism rather than architectural novelty alone [1,2]. The hyperparameters in Table 5 were fixed a priori as practical baseline settings commonly used for sparse molecular-fingerprint classification: moderate tree depth or leaf count, shrinkage for boosting, subsampling/feature subsampling for regularization and class-weighting to address imbalance. No target-specific hyperparameter search was performed, and the test sets were not used for model selection. This choice was made to avoid turning the study into a target-tuned leaderboard and to keep the comparison centred on split realism, similarity baselines, calibration and downstream triage. ECFP4 was chosen because it is directly compatible with the nearest-active Tanimoto baseline and keeps the main comparison anchored to a transparent, widely used ligand representation. To check whether the main target-level interpretation was narrowly dependent on this representation, we also ran a compact scaffold-split sensitivity analysis with ECFP6 fingerprints (Morgan radius 3, 2048 bits) using the same model family and seed. Both random and scaffold splits were evaluated. To manage runtime during exploratory development while preserving all actives, each target was capped at 80,000 rows in the first repeated-seed benchmark. This cap affected only the PKM2 inactive pool; the cleaned MAPK1 set contained fewer than 80,000 rows. To check whether the PKM2 scaffold-split conclusion was an artefact of this cap, we repeated the seed-42 PKM2 scaffold-split run on the full cleaned PKM2 set for the main tree models and compared it with the full-data nearest-active similarity baseline (Supplementary Table S4). The main model settings used throughout the study are summarized in Table 5.

Performance was evaluated using average precision (AP), ROC-AUC, enrichment factor at 1% of the ranked list (EF1%) and BEDROC20. Accuracy was not used as a primary metric because both targets are highly imbalanced and virtual screening is an early-recognition problem [5].

### 4.5. Computational Environment

All analyses were executed in a dedicated conda environment on a MacBook Pro equipped with an Apple M3 Max chip (16 CPU cores; 12 performance and 4 efficiency cores) and 64 GB unified memory. The main software stack included Python 3.12.8, RDKit 2025.09.5, scikit-learn 1.7.2, LightGBM 4.6.0 and XGBoost 3.1.2. Structure-based triage was performed with AutoDock Vina 1.2.5, with receptor and ligand preparation handled through the same environment together with Meeko 0.7.1 and RDKit 2025.09.5. This information is reported to make the runtime and implementation context explicit rather than to suggest hardware-dependent claims.

### 4.6. Probability Calibration and Uncertainty

For scaffold-split LightGBM and XGBoost models, an internal calibration set was reserved from the training data. Specifically, after the external 80:20 train–test split was created, 20% of the scaffold-training partition was held out for isotonic calibration, while the remaining 80% was used to fit the base model. Isotonic regression was used to calibrate raw model scores, following the broader literature showing that strong discriminative learners often require explicit probability calibration before their outputs are interpreted probabilistically [27]. Calibration quality was evaluated using Brier score, log-loss and expected calibration error with 10 bins (ECE10). A two-model calibrated ensemble was then summarized by its mean calibrated score and model-disagreement standard deviation. We treated this disagreement term as a pragmatic uncertainty proxy for ranking support rather than as a formal Bayesian quantity, in line with recent chemistry-facing discussions that distinguish useful uncertainty characterization from overconfident point prediction [22,23]. These quantities were used for downstream prioritization rather than raw classifier scores alone.

### 4.7. ADMET-Aware Prioritization

Candidate compounds were first filtered using soft drug-likeness constraints based on molecular weight, logP, TPSA, Lipinski violations and Veber criteria. ADMET-AI was then applied to scaffold-diverse top-ranked candidates [28]. ADMET outputs were converted into a support score that combined absorption-related predictions, quantitative estimate of drug-likeness (QED), solubility, median lethal dose (LD50), structural alerts and toxicity-burden indicators. These values were used as computational risk signals, not as experimental safety claims. Accordingly, the term ADMET-aware is used in this manuscript to mean that predicted pharmacokinetic and toxicity-related liabilities were considered during prioritization; it does not imply that absorption, distribution, metabolism, excretion, toxicity, pharmacokinetic behaviour or compound safety were experimentally confirmed.

### 4.8. Molecular Docking and Pose-Level Interaction Fingerprints

The top ADMET-aware candidates were docked with AutoDock Vina to add a molecular-level structural layer to the final prioritization stage. PKM2 docking used the local LIT-PCBA 4G1N receptor files, and MAPK1 docking used the local 4QTE receptor files. Receptors and ligands were prepared with Meeko, and ligand conformers were generated with RDKit ETKDG followed by force-field optimization. The Vina search boxes were derived from the corresponding LIT-PCBA pocket files. MAPK1/4QTE was used as the ligand-bound ERK2 receptor conformation supplied in the LIT-PCBA benchmark; no alternative active/inactive ERK2 conformational ensemble was docked, so the MAPK1 docking results should be interpreted as conditional on the 4QTE pocket geometry. During receptor preparation, alternate location A was retained for chain-A residues VAL39, ASN144, LYS164, ARG172, HIS180, GLU186, PRO226, LEU244, ASP251, ASN257, LEU258, ASN276, SER284, THR295, ASP336 and GLU350. Each candidate ligand was represented by one RDKit ETKDGv3 conformer generated from the canonical SMILES string using a fixed seed, followed by MMFF optimization where possible and UFF optimization as a fallback. Ligand PDBQT files were then prepared with Meeko using its default Gasteiger charge assignment; no separate pH-specific protonation or tautomer enumeration step was applied. The local LIT-PCBA receptor files used for docking are protein-coordinate files and did not retain crystallographic waters or the small-molecule cofactors present in the original PDB deposition; these species were therefore not included in the Vina receptor PDBQT files. For PKM2, this simplification may affect local docking scores or contact patterns in the allosteric activator pocket if structural waters contribute to ligand stabilization; therefore, PKM2 docking scores are interpreted as water-free triage scores rather than definitive binding-mode evidence. For PKM2, the 4G1N pocket was not treated as a blind whole-protein search. It was the LIT-PCBA ligand-defined pocket around the co-crystallized activator NZT, corresponding to an allosteric activator pocket rather than the orthosteric catalytic site or a de novo FBP-site search. For reproducibility, the final Vina settings were exhaustiveness = 16, num_modes = 10 and cpu = 4. The PKM2/4G1N box was centred at (1.558, −12.069, 46.389) Å with dimensions 32.641 × 40.059 × 35.962 Å; the MAPK1/4QTE box was centred at (22.535, 71.198, 23.695) Å with dimensions 34.622 × 40.653 × 32.936 Å. As an internal protocol check, the co-crystallized reference ligands were re-docked into the same receptor boxes and compared with their crystallographic coordinates by direct heavy-atom RMSD. PKM2/4G1N was recovered by the top-ranked redocked pose with an RMSD of 0.54 Å. For MAPK1/4QTE, a near-native pose was generated among the Vina modes (minimum RMSD 1.32 Å, mode 9), although the top-scoring pose had a higher RMSD of 11.12 Å. This target-dependent redocking behaviour reinforces the conservative interpretation used throughout the manuscript: docking was retained as a structural triage and pose-sanity layer, not as proof of binding affinity. The docking setup and redocking check are summarized in Table 6. This structure-based step was used as a downstream triage layer rather than as a standalone claim of binding, in line with practical docking workflows that treat docking as one component of a broader virtual-screening pipeline [7,24].

For pose sanity checking, the first Vina pose for each candidate was parsed directly from PDBQT coordinates. Residue-level contacts within 4.5 Å were extracted and categorized as hydrophobic, aromatic, polar/hydrogen-bond-like, halogen-polar or close contacts using atom-type and residue-class heuristics. This interaction fingerprint was used as a molecular-level check on whether high-ranked candidates occupied the intended pockets and shared coherent target-specific contact patterns. Final structure-aware consensus ranking was computed within each target over the scaffold-diverse top-30 docked candidates. The score combined min–max-scaled calibrated ensemble activity, ADMET support, docking support and inverse toxicity burden as $S_{\mathrm{consensus}}=0.35S_{\mathrm{activity}}+0.25S_{\mathrm{ADMET}}+0.25S_{\mathrm{docking}}+0.15\left(1-S_{\mathrm{toxicity}}\right)$, where docking support was the inverted min–max-scaled Vina score so that more negative Vina scores received higher support. The LIT-PCBA label was not used in this final ranking; it was retained only for retrospective benchmark auditing. Thus, label-0 molecules in the final list should be read as computationally prioritized benchmark negatives, not as experimentally confirmed hits or as claims of true activity.

## 5. Conclusions

We developed a leakage-aware, uncertainty-calibrated and multi-objective CADD workflow for PKM2 and MAPK1 virtual screening. The study shows that benchmark enrichment is most informative when it is interpreted alongside scaffold splits, explicit similarity baselines and calibration quality rather than in isolation. It also shows that molecular docking and residue-level contact analysis can add a useful structural layer to computational prioritization when they are used with restraint and interpreted together with ligand-based evidence.

PKM2 emerged as the strongest target branch, with coherent support across scaffold-aware machine learning, calibration, ADMET profiling, docking and recurrent pocket-contact patterns. MAPK1 provided a useful contrast case in which biological relevance remained high but ligand-based ML superiority was less pronounced, making downstream structural and ADMET-aware filtering more important. This difference between the two targets is not a weakness of the manuscript; it helps define where the workflow appears most informative and where it should be applied more cautiously.

The main value of the study lies in making each decision layer visible. Rather than presenting a single best score as a surrogate for discovery success, the manuscript organizes candidate prioritization as a sequence of interpretable steps, from curation and benchmark realism to molecular-level structural checks. In that sense, the work offers a transparent computational framework for cancer-focused drug discovery while keeping its claims aligned with the limits of experimentally unvalidated evidence.

This point is especially relevant for realistic screening campaigns in which chemical redundancy, ranking instability and downstream attrition can obscure whether a candidate list is genuinely informative. By separating those issues instead of collapsing them into a single performance number, the workflow remains easier to audit, easier to extend and easier to interpret in future computational or experimental follow-up studies.

## Figures and Tables

**Table 1:** Target-level summary of the raw and cleaned LIT-PCBA subsets used in this study.

Target	Raw Rows	Clean Rows	Actives	Inactives	Duplicates Removed	Label Conflicts Removed
MAPK1	62,937	61,990	302	61,688	941	6
PKM2	246,069	245,322	546	244,776	747	0

**Table 2:** Similarity baseline performance and best first-pass scaffold-aware ML results for PKM2 and MAPK1.

Target	Setting	AP	ROC-AUC	EF1%	BEDROC20
PKM2	Similarity, random split	0.1020	0.8101	33.92	0.4656
PKM2	Similarity, scaffold split	0.0063	0.7017	10.89	0.2448
PKM2	Best ML, scaffold split (XGBoost)	0.1603	0.7770	31.17	0.4338
MAPK1	Similarity, random split	0.0281	0.6959	11.66	0.2298
MAPK1	Similarity, scaffold split	0.0507	0.7490	21.15	0.3408
MAPK1	Best ML, scaffold split (LightGBM)	0.0722	0.7232	19.23	0.2908

**Table 3:** Best repeated-seed model performance for each target and split (mean ± standard deviation over five seeds).

Target	Split	Best Model	AP	EF1%	BEDROC20
PKM2	Random	LightGBM	0.326 ± 0.029	40.73 ± 3.70	0.568 ± 0.024
PKM2	Scaffold	XGBoost	0.157 ± 0.024	30.01 ± 0.97	0.435 ± 0.018
MAPK1	Random	XGBoost	0.034 ± 0.011	10.33 ± 3.42	0.239 ± 0.020
MAPK1	Scaffold	LightGBM	0.076 ± 0.010	17.86 ± 2.41	0.316 ± 0.024

**Table 4:** Consensus-ranked docked candidates per target after integrating calibrated activity, ADMET support, inverse toxicity burden and docking support. Labels are retrospective LIT-PCBA benchmark labels and were not used to compute the consensus score.

Target	Candidate	Label	Calibrated Score	ADMET Support	Toxicity Burden	Vina Score (kcal/mol)	Consensus Score
PKM2	PKM2-r1	1	0.834	0.746	0.344	−9.534	0.839
PKM2	PKM2-r2	1	0.768	0.702	0.371	−10.240	0.816
PKM2	PKM2-r3	1	0.768	0.733	0.315	−9.543	0.816
PKM2	PKM2-r4	1	0.532	0.650	0.381	−10.290	0.645
PKM2	PKM2-r5	0	0.211	0.805	0.389	−9.589	0.570
MAPK1	MAPK1-r1	0	0.065	0.764	0.410	−9.068	0.872
MAPK1	MAPK1-r2	0	0.062	0.705	0.458	−9.929	0.829
MAPK1	MAPK1-r3	0	0.062	0.709	0.448	−8.915	0.755
MAPK1	MAPK1-r4	1	0.065	0.640	0.521	−8.909	0.709
MAPK1	MAPK1-r5	0	0.062	0.626	0.484	−9.093	0.681

**Table 5:** Principal model and workflow settings used in the ligand-based benchmark, calibration and docking stages.

Stage	Component	Key Settings
Ligand representation	ECFP4 fingerprints	Morgan radius 2 (diameter 4), 2048 bits; circular fingerprint baseline used for similarity and ML models
Representation sensitivity	ECFP6 fingerprints	Morgan radius 3, 2048 bits; scaffold-split sensitivity check using the same model family and seed
Data splitting	Random and scaffold split	Same target-level clean tables; repeated-seed analysis over seeds 11, 23, 42, 67 and 101
Exploratory benchmark	Row cap	Maximum 80,000 rows per target during repeated-seed benchmark; all actives retained
Similarity baseline	Nearest-active Tanimoto	Maximum similarity to active molecules in the corresponding training fold
Logistic regression	sklearn	class_weight=balanced, solver=saga, penalty=l2, C=1.0, max_iter=1000
Random forest	sklearn	n_estimators=300, min_samples_leaf=2, class_weight=balanced_subsample
LightGBM	Weighted gradient boosting	n_estimators=600, learning_rate=0.03, num_leaves=31, subsample=0.8, colsample_bytree=0.7
XGBoost	Weighted gradient boosting	n_estimators=600, max_depth=5, learning_rate=0.03, subsample=0.8, colsample_bytree=0.7, tree_method=hist
Class imbalance handling	Tree models	scale_pos_weight set from the training split for calibrated LightGBM and XGBoost runs
Calibration	Isotonic regression	Internal calibration split reserved from the scaffold-training partition; ECE10, Brier score and log-loss reported
Uncertainty proxy	Calibrated ensemble disagreement	Mean calibrated score plus standard deviation across calibrated LightGBM/XGBoost predictions
Docking	AutoDock Vina	cpu=4; receptor-specific pocket box from LIT-PCBA pocket files; best-ranked pose retained for summary
Ligand 3D generation	RDKit ETKDG/MMFF	ETKDGv3 conformer generation followed by force-field optimization before docking preparation

**Table 6:** Docking box settings and reference-ligand redocking validation. RMSD values were calculated against the prepared crystallographic ligand coordinates using heavy atoms.

Target	PDB	Center X	Center Y	Center Z	Size X	Size Y	Size Z	Redocking RMSD
PKM2	4G1N	1.558	−12.069	46.389	32.641	40.059	35.962	0.54 Å (top pose)
MAPK1	4QTE	22.535	71.198	23.695	34.622	40.653	32.936	1.32 Å (minimum; mode 9)

## Data Availability

The LIT-PCBA target subsets used in this study are publicly available from the LIT-PCBA benchmark distribution. To support review and reproducibility, the submission package includes processed target tables, consensus-candidate SMILES, train/test output files, repeated-seed results, ADMET prediction outputs, docking configuration files, prepared receptor and ligand PDBQT files, Vina logs, docked poses, redocking validation outputs and analysis scripts as supplementary material. These files are itemized in Supplementary Table S3. The complete reproducibility package is available through the public GitHub repository at https://github.com/drferhatu/Leakage-Aware-Drug-Discovery-Workflow-for-PKM2-and-MAPK1.git (accessed on 20 May 2026) and archived on Zenodo at https://doi.org/10.5281/zenodo.20342954.

## Supplementary Materials

The following supporting information can be downloaded at: https://www.mdpi.com/article/10.3390/ijms27114751/s1.
