# Supplementary material for: A Leakage-Aware Drug Discovery Workflow for PKM2 and MAPK1 Integrating Scaffold Validation, Molecular Docking and Structural Triage
Source: Int J Mol Sci. 2026 May 25;27(11):4751. doi: 10.3390/ijms27114751 (PMC13257026; doi:10.3390/ijms27114751)
Supplement: Supplementary file 1 [file ijms-27-04751-s001.zip › ijms-4310641-supplementary.pdf]

Supplementary Materials

Supplementary Figure S1

Shared-axis ADMET-aware prioritization plot for the scaffold-diverse candidates. In contrast to the target-specific x-axis scaling used in the main figure, this supplementary view uses a common calibrated-score range for PKM2 and MAPK1, making the narrow absolute calibrated-score range of the MAPK1 shortlist directly visible.

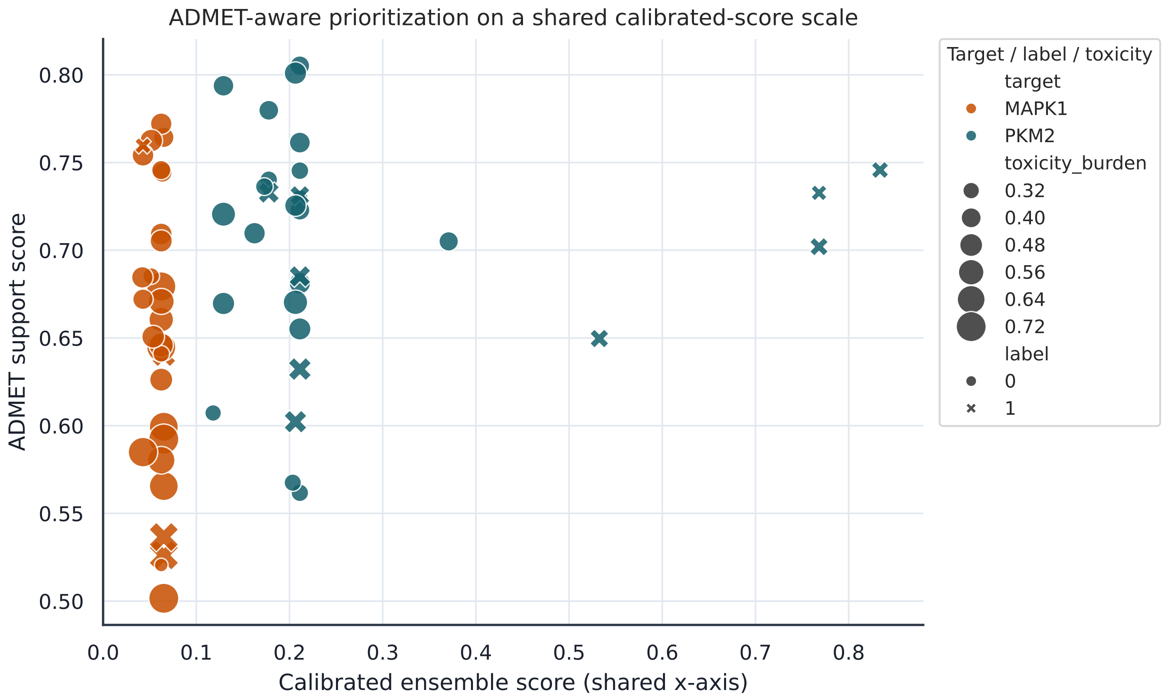

Supplementary Table S1

The most frequent residue-level pocket contacts across the top five consensus-ranked ligands for PKM2 and MAPK1. Frequencies are reported as the number of ligands in the top-five consensus shortlist that contacted a given residue. Mean and minimum distances are reported in Å. The **Close** column is not additive with the specific interaction-type columns; it reports residue contacts within the 4.5 Å cutoff that were not otherwise classified by the hydrophobic, aromatic, hydrogen-bond-like, polar or halogen-contact rules.

Target	Residue	Freq.	Rows	Mean d_{min}	Min d_{min}	Hydroph.	Arom.	H-bond-like	Polar	Halogen	Close
MAPK1	GLUA33	5/5	5	3.648	3.129	0	0	1	3	0	5
MAPK1	ILEA31	5/5	5	3.611	3.360	4	0	0	0	0	5
MAPK1	LEUA156	5/5	5	3.388	3.148	4	0	0	0	0	5
MAPK1	VALA39	5/5	5	3.562	3.133	5	0	0	0	0	5
MAPK1	ALAA52	4/5	4	3.657	3.395	4	0	0	0	0	3
MAPK1	ASPA106	4/5	4	3.764	3.443	0	0	1	3	0	3
MAPK1	ASPA111	4/5	4	3.403	3.150	0	0	0	4	1	3
MAPK1	ASPA167	4/5	4	3.305	2.515	0	0	2	4	0	4
MAPK1	CYSA166	4/5	4	3.758	2.848	0	0	0	2	0	4
MAPK1	GLNA105	4/5	4	3.295	2.918	0	0	1	4	0	4
MAPK1	GLYA32	4/5	4	3.955	3.747	0	0	0	0	0	4
MAPK1	ILEA84	4/5	4	3.783	3.129	2	0	0	0	0	3
PKM2	ASPB354	5/5	5	3.624	3.216	0	0	1	3	0	4
PKM2	LYSA311	5/5	5	3.311	2.680	0	0	1	4	0	4
PKM2	LYSB311	5/5	5	2.542	1.910	0	0	4	5	0	5
PKM2	META30	5/5	5	3.803	3.412	2	0	0	0	0	5
PKM2	PHEA26	5/5	5	3.573	3.432	5	5	0	0	0	5
PKM2	PHEB26	5/5	5	3.583	3.498	5	5	0	0	0	5
PKM2	ILEA389	4/5	4	3.324	3.122	2	0	0	0	0	4
PKM2	ILEB389	4/5	4	3.693	3.474	2	0	0	0	0	4
PKM2	LEUA353	4/5	4	3.160	2.435	4	0	1	0	0	4
PKM2	LEUB353	4/5	4	3.622	3.550	4	0	0	0	0	4
PKM2	TYRA390	4/5	4	2.075	1.798	1	4	3	4	1	4
PKM2	TYRB390	4/5	4	2.430	1.901	0	3	2	4	0	4

Supplementary Table S2

The top five consensus-ranked docked candidates for each target after integrating calibrated activity, ADMET support, toxicity burden and Vina docking support. The Label column reports the retrospective LIT-PCBA benchmark label, not experimental validation in this study.

Target	Rank	SMILES	Label	Calib.	ADMET	Toxic.	Vina	Consensus
MAPK1	1	O=C(Nc1ccc(F)c(F)c1)c1ccc(F)c(S(=O)(=O)N2CCOCC2)c1	0	0.065	0.764	0.410	-9.068	0.872
MAPK1	2	Clc1ccccc1-c1ccc2ncnc(N3CC[NH2+]CC3)c2c1	0	0.062	0.705	0.458	-9.929	0.829
MAPK1	3	COc1ccccc1-c1ccc2ncnc(N3CC[NH2+]CC3)c2c1	0	0.062	0.709	0.448	-8.915	0.755
MAPK1	4	COc1ccc(C2=Nn3c(nnc3-c3ccccc3OC)SC2)cc1OC	1	0.065	0.640	0.521	-8.909	0.709
MAPK1	5	COc1ccccc1CNc1ncnc2ccc(-c3ccccc3C[NH+](C)C)cc12	0	0.062	0.626	0.484	-9.093	0.681
PKM2	1	Cc1ccc(NS(=O)(=O)c2ccc3c(c2)oc(=O)n3C)cc1F	1	0.834	0.746	0.344	-9.534	0.839
PKM2	2	Cc1cc2c(cc1S(=O)(=O)Nc1ccc(F)cc1)n(C)c(=O)c(=O)n2C	1	0.768	0.702	0.371	-10.240	0.816
PKM2	3	Cn1c(=O)oc2cc(S(=O)(=O)Nc3ccccc3)ccc21	1	0.768	0.733	0.315	-9.543	0.816
PKM2	4	COc1cccc(NS(=O)(=O)c2ccc3c(c2)n(C)c(=O)c(=O)n3C)c1	1	0.532	0.650	0.381	-10.290	0.645
PKM2	5	Cc1ccc(S(=O)(=O)N2CCN(C(=O)c3ccco3)CC2)cc1	0	0.211	0.805	0.389	-9.589	0.570

Supplementary Table S3

Reproducibility manifest for the revised manuscript. File paths refer to the project archive that accompanies the revision and will be mirrored in the public repository and Zenodo archive.

Item	File(s)	Purpose
Processed target tables	`scripts/data/processed/lit_pcba_pkm2_mapk1_clean.csv`; `scripts/data/processed/lit_pcba_pkm2_mapk1_clean_summary.csv`	Cleaned canonical-SMILES tables and target-level counts.
Train/test and seed-dependent outputs	`scripts/data/processed/ml_baseline_results.csv`; `scripts/data/processed/repeated_seed/repeated_seed_ml_results.csv`	Model outputs for random/scaffold splits and repeated seeds.
Similarity baseline	`scripts/data/processed/similarity_baseline_results.csv`	Nearest-active Tanimoto baseline results for random and scaffold splits.
Representation sensitivity	`scripts/data/processed/ml_baseline_ecfp6_sensitivity_results.csv`	Additional ECFP6 scaffold-split sensitivity check.
Calibration outputs	`scripts/data/processed/calibration/calibration_metrics.csv`; `scripts/data/processed/calibration/calibrated_test_predictions.csv`	Calibration metrics and calibrated test predictions.
ADMET outputs	`scripts/data/processed/admet/combined_diverse_top30_admet_predictions.csv`; `scripts/data/processed/admet/combined_diverse_top30_admet_annotated.csv`	ADMET-AI predictions and annotated top-30 candidate tables.
Top candidate SMILES and consensus tables	`MDPI_Latex/supplementary/supp_table_s2_top_consensus_candidates.csv`; `MDPI_Latex/supplementary/supp_table_s5_full_top30_consensus.csv`	Consensus-ranked candidate SMILES, retrospective labels and final prioritization scores.
Docking inputs and logs	`scripts/docking_top30/configs/`; `scripts/docking_top30/receptors/`; `scripts/docking_top30/ligands_pdbqt/`; `scripts/docking_top30/results/logs/`	Vina configuration files, prepared receptors/ligands and docking logs for the expanded top-30 docking analysis.
Docking poses and contact fingerprints	`scripts/docking_top30/results/poses/`; `scripts/docking/interactions/pose_residue_contacts.csv`	Docked top-30 poses and residue-level contact tables for the consensus-ranked ligands.
Redocking validation	`scripts/docking/redocking_validation/redocking_validation_summary.csv`; `scripts/docking/redocking_validation/redocking_rmsd_by_mode.csv`	Reference-ligand redocking RMSD summaries.
Revision figures	`MDPI_Latex/figs/generated/fig16_docking_consensus_panel.png`; `MDPI_Latex/figs/generated/fig18_candidate_2d_structures.png`	Updated structure-aware triage and 2D candidate-structure figures.
Analysis scripts	`scripts/*.py`	Scripts used for curation, model training, calibration, prioritization, docking analysis and figure generation.

Supplementary Table S4

Full-data PKM2 scaffold-split sensitivity check for seed 42. The repeated-seed benchmark in the main text used an 80,000-row per-target cap while retaining all actives. This supplementary check restores the complete cleaned PKM2 inactive pool and compares the full-data nearest-active similarity baseline with full-data tree-model runs on the same scaffold split.

Method	Train rows	Test rows	Train actives	Test actives	AP	EF1%	BEDROC20
Nearest-active Tanimoto	196,214	49,108	491	55	0.0063	10.89	0.245
LightGBM	196,214	49,108	491	55	0.0069	16.33	0.257
XGBoost	196,214	49,108	491	55	0.0061	14.52	0.263

Supplementary Table S5

Full scaffold-diverse top-30 consensus table for each target after ADMET-aware prioritization and Vina docking. The Label column reports the retrospective LIT-PCBA benchmark label and is included to audit false-positive risk in the computational ranking. The first five rows per target deliberately overlap with Supplementary Table S2; S2 is retained as the narrative top-five summary, whereas S5 provides the complete top-30 audit.

Target	Rank	SMILES	Label	Calib.	ADMET	Toxic.	Vina	Consensus
MAPK1	1	O=C(Nc1ccc(F)c(F)c1)c1ccc(F)c(S(=O)(=O)N2CCOCC2)c1	0	0.065	0.764	0.410	-9.068	0.872
MAPK1	2	Clc1ccccc1-c1ccc2ncnc(N3CC[NH2+]CC3)c2c1	0	0.062	0.705	0.458	-9.929	0.829
MAPK1	3	COc1ccccc1-c1ccc2ncnc(N3CC[NH2+]CC3)c2c1	0	0.062	0.709	0.448	-8.915	0.755
MAPK1	4	COc1ccc(C2=Nn3c(nnc3-c3ccccc3OC)SC2)cc1OC	1	0.065	0.640	0.521	-8.909	0.709
MAPK1	5	COc1ccccc1CNc1ncnc2ccc(-c3ccccc3C[NH+](C)C)cc12	0	0.062	0.626	0.484	-9.093	0.681
MAPK1	6	Cn1c(=O)[nH]c(=O)c2c1nc(Br)n2Cc1ccccc1Cl	0	0.062	0.746	0.380	-7.164	0.671
MAPK1	7	CCn1c(SCC(N)=O)nc2ccccc2c1=O	0	0.062	0.772	0.438	-7.094	0.671
MAPK1	8	C=CCSc1nc2c(c(=O)[nH]c(=O)n2C)n1CC	0	0.064	0.744	0.391	-6.839	0.658
MAPK1	9	COc1ccc(-c2nnc3n2N=C(c2ccccc2)CS3)cc1OC	0	0.062	0.646	0.518	-8.464	0.637
MAPK1	10	N#Cc1cccc(-c2nc(NCc3ccccc3)c3ccccc3n2)c1	1	0.065	0.526	0.716	-9.975	0.624
MAPK1	11	COc1ccc(C[NH+]2CCN(c3cccc(C)c3C)CC2)cc1OC	0	0.062	0.641	0.323	-7.496	0.620
MAPK1	12	Cc1cccc(CNc2ccnc(-c3cccc(C#N)c3)n2)c1	0	0.065	0.566	0.676	-9.268	0.617
MAPK1	13	CNc1ncnc2ccc(-c3ccccc3OC)cc12	0	0.062	0.679	0.690	-8.526	0.617
MAPK1	14	N#Cc1cccc(-c2nccc(NCc3ccccc3)n2)c1	0	0.065	0.599	0.681	-8.839	0.613
MAPK1	15	COc1ccccc1-c1ccc2ncnc(NCc3ccccc3)c2c1	1	0.065	0.537	0.708	-9.061	0.563
MAPK1	16	COc1ccc(-c2nc(C#N)c(NCCC[NH+](C)C)o2)cc1OC	0	0.062	0.660	0.530	-7.233	0.549
MAPK1	17	COc1ccccc1N1CC[NH+](Cc2cccc(OC)c2OC)CC1	0	0.052	0.685	0.321	-8.056	0.543
MAPK1	18	CC[NH+](CC)CCCNc1oc(-c2ccc(OC)c(OC)c2)nc1C#N	0	0.062	0.671	0.573	-7.134	0.536
MAPK1	19	COCCNc1ncnc2ccc(-c3ccccc3OC)cc12	0	0.062	0.645	0.683	-7.826	0.531
MAPK1	20	COc1cccc(-c2nc(NCc3ccccc3OC)c3ccccc3n2)c1	0	0.065	0.502	0.720	-9.025	0.524
MAPK1	21	COc1cccc(-c2cncnc2NCc2cccc(C)c2)c1	0	0.062	0.580	0.627	-8.182	0.519
MAPK1	22	COCCNS(=O)(=O)c1cc(-c2nn(C)c(=O)c3ccccc23)ccc1C	0	0.054	0.651	0.475	-8.295	0.511
MAPK1	23	COCCNc1nc(-c2cccc(OC)c2)nc2ccccc12	0	0.065	0.592	0.720	-7.669	0.500
MAPK1	24	CC[NH+](CC)CCSc1nc(O)c(-c2ccccc2)c(O)n1	0	0.062	0.521	0.263	-7.083	0.495
MAPK1	25	CC(=O)N(C)c1nc(-c2ccc(Cl)cc2)cs1	0	0.052	0.763	0.471	-6.964	0.478
MAPK1	26	CCc1nc(N)c(C#N)c(-c2ccc(OC)c(OC)c2)c1C	0	0.043	0.754	0.445	-8.202	0.442
MAPK1	27	COc1ccc(S(=O)(=O)c2ccc(OC)c(OC)c2)cc1OC	1	0.043	0.760	0.322	-6.952	0.388
MAPK1	28	C=CCSc1nc(N)c(C#N)c(-c2ccccc2)c1C#N	0	0.043	0.672	0.410	-7.652	0.334

Target	Rank	SMILES	Label	Calib.	ADMET	Toxic.	Vina	Consensus
MAPK1	29	`CCN(CC)S(=O)(=O)c1ccc(C(=O)Nc2ccc3c(c2)OCCO3)cc1`	0	0.042	0.685	0.438	-7.734	0.333
MAPK1	30	`Nc1ccc(-c2nc3c(Cl)cc(N)cc3[nH]2)cc1`	0	0.043	0.585	0.699	-7.542	0.150
PKM2	1	`Cc1ccc(NS(=O)(=O)c2ccc3c(c2)oc(=O)n3C)cc1F`	1	0.834	0.746	0.344	-9.534	0.839
PKM2	2	`Cc1cc2c(cc1S(=O)(=O)Nc1ccc(F)cc1)n(C)c(=O)c(=O)n2C`	1	0.768	0.702	0.371	-10.240	0.816
PKM2	3	`Cn1c(=O)oc2cc(S(=O)(=O)Nc3ccccc3)ccc21`	1	0.768	0.733	0.315	-9.543	0.816
PKM2	4	`COc1cccc(NS(=O)(=O)c2ccc3c(c2)n(C)c(=O)c(=O)n3C)c1`	1	0.532	0.650	0.381	-10.290	0.645
PKM2	5	`Cc1ccc(S(=O)(=O)N2CCN(C(=O)c3ccco3)CC2)cc1`	0	0.211	0.805	0.389	-9.589	0.570
PKM2	6	`CCC(CC)C(=O)N1N=C(c2ccc(NS(C)(=O)=O)cc2)CC1c1ccccc1`	0	0.206	0.725	0.441	-10.250	0.517
PKM2	7	`CCc1ccc(OC)c(S(=O)(=O)N2CCN(C(=O)c3ccco3)CC2)c1`	0	0.178	0.780	0.396	-9.113	0.474
PKM2	8	`N#Cc1ccccc1S(=O)(=O)Nc1ccc2c(c1)OCCO2`	0	0.173	0.736	0.344	-9.187	0.471
PKM2	9	`O=S(=O)(c1ccccc1F)N1CCc2ccccc2C1`	0	0.129	0.794	0.418	-9.282	0.466
PKM2	10	`CCNC(=S)N1CCN(S(=O)(=O)c2ccc(C)c(C)c2)CC1`	0	0.211	0.761	0.423	-9.190	0.461
PKM2	11	`Cc1ccc(S(=O)(=O)Nc2cccnc2)cc1C`	0	0.211	0.730	0.313	-8.587	0.444
PKM2	12	`COc1ccc(S(=O)(=O)N2CCc3cc(OC)c(OC)cc3C2)cc1`	1	0.178	0.733	0.444	-9.544	0.436
PKM2	13	`COc1ccc(C)cc1S(=O)(=O)Nc1ccccc1`	0	0.211	0.745	0.346	-8.287	0.406
PKM2	14	`Cc1cc(C)cc(NS(=O)(=O)c2cccs2)c1`	0	0.371	0.705	0.385	-8.049	0.390
PKM2	15	`COc1ccc(S(=O)(=O)Nc2cccnc2)c(C)c1C`	0	0.178	0.740	0.340	-8.297	0.389
PKM2	16	`CCS(=O)(=O)N1CCN(S(=O)(=O)c2ccc(F)cc2)CC1`	0	0.206	0.801	0.464	-8.259	0.374
PKM2	17	`COc1ccc(S(=O)(=O)N2CC[NH+](Cc3cccc(Cl)c3)CC2)cc1`	0	0.204	0.568	0.336	-9.620	0.363
PKM2	18	`O=S(=O)(Nc1ccc2c(c1)OCO2)c1ccccc1`	0	0.211	0.723	0.400	-8.415	0.357
PKM2	19	`Cc1ccc(NS(=O)(=O)c2ccccc2[N+](=O)[O-])cc1C`	0	0.211	0.655	0.460	-9.499	0.357
PKM2	20	`COc1ccc(S(=O)(=O)Nc2ccc3c(c2)OCO3)cc1C`	0	0.211	0.681	0.439	-9.080	0.355
PKM2	21	`Cc1ccc(S(=O)(=O)NCc2ccc3c(c2)OCO3)cc1C`	0	0.206	0.670	0.526	-9.807	0.355
PKM2	22	`CCC(CC)C(=O)N1N=C(c2ccc(NS(C)(=O)=O)cc2)CC1c1ccc(C)cc1`	0	0.163	0.710	0.442	-9.042	0.355
PKM2	23	`O=C(CSc1ccc(-c2ccco2)nn1)Nc1ccccc1`	1	0.211	0.685	0.458	-9.050	0.343
PKM2	24	`COc1cccc(C[NH+]2CCN(S(=O)(=O)c3ccc(Br)cc3)CC2)c1`	0	0.211	0.562	0.339	-9.342	0.330
PKM2	25	`O=S(=O)(NCc1ccc2c(c1)OCO2)c1ccc(F)cc1`	0	0.129	0.721	0.518	-9.239	0.317
PKM2	26	`N#CCc1ccc(NS(=O)(=O)c2cccs2)cc1`	1	0.211	0.731	0.415	-8.008	0.314
PKM2	27	`CNS(=O)(=O)c1cc2c3c(cccc3c1)C(=O)NC2=O`	1	0.206	0.602	0.488	-9.121	0.242
PKM2	28	`COc1cccc(NC(=O)CSc2ccc(-c3ccco3)nn2)c1`	1	0.211	0.632	0.495	-8.657	0.222
PKM2	29	`COC(=O)CC[NH+]1CCN(S(=O)(=O)c2cccc(Br)c2)CC1`	0	0.118	0.607	0.319	-8.017	0.210
PKM2	30	`Cc1ncc([N+](=O)[O-])n1S(=O)(=O)c1cc(Br)ccc1Br`	0	0.129	0.670	0.466	-7.849	0.158
